# Supplementary material for: Immersive virtual environments and embodied agents for e-learning applications
Source: PeerJ Comput Sci. 2020 Nov 16;6:e315. doi: 10.7717/peerj-cs.315 (PMC7924662; doi:10.7717/peerj-cs.315)
Supplement: Supplemental Information 2 — Contains information about a made-up alien species which participants had to learn about during the study. [file peerj-cs-06-315-s002.pdf]

The Crixwin Stages of Development

1: Negirans Stage

2: Furlagane Stage

3: Levorg Stage

4: Gresians Stage

5: Arderlons Stage

6: Impons Stage

7: Borgavari Stage

Stage 1: Negirans Stage

Gas is secreted into a type of hole in the ground by a parent Crixwin

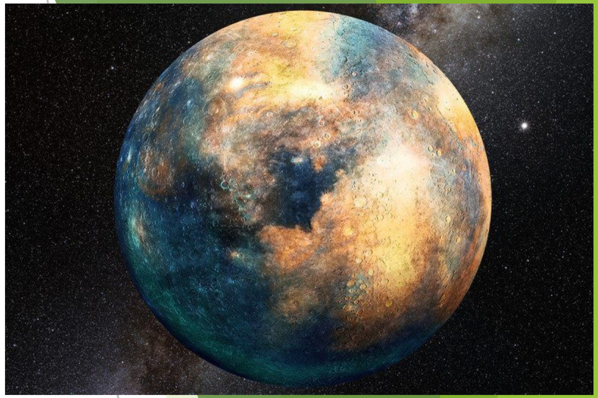

- After **17 Earth months**, the pregnant **adult Crixwin secretes a gaseous substance into** naturally occurring **underground caverns (kurins) found on Planet Alun's surface**

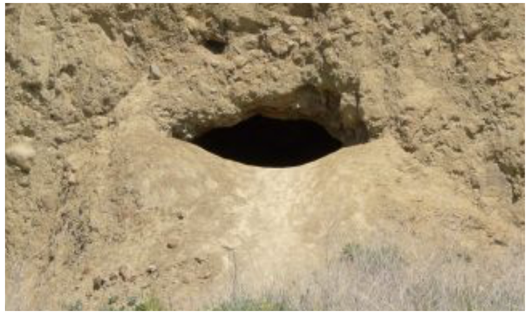

- **Kurins** (small, spherical caverns which form in the soil) must be **found using a form of echolocation** which responds to vibrations travelling through the soil

- **Optimal kurins will be large** but strong enough to maintain a consistent structure, as larger space allows for larger and more developed Crixwins, but too large and the kurin will collapse in on itself as the Crixwin develops

- The vapour secreted contains all the necessary chemicals for the construction of tissues necessary for stage 2

How are Kurins formed?

- Kurins are formed by the life cycle of **a type of flora (lo'roon)** which will **form from a spore landing on the surface soil, spreading into the ground and creating a large spherical sac** which produces more spores

- When **spores are released the sac dissolves leaving the spherical shape** – the only thing left behind are the distinctive vents from which the spores are released

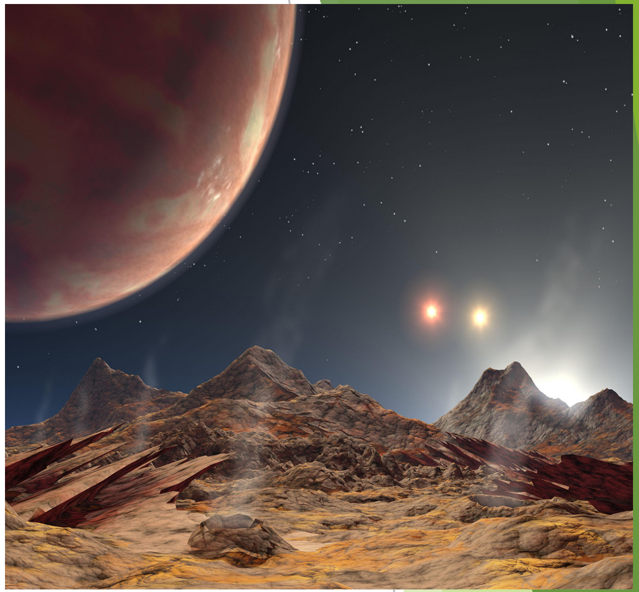

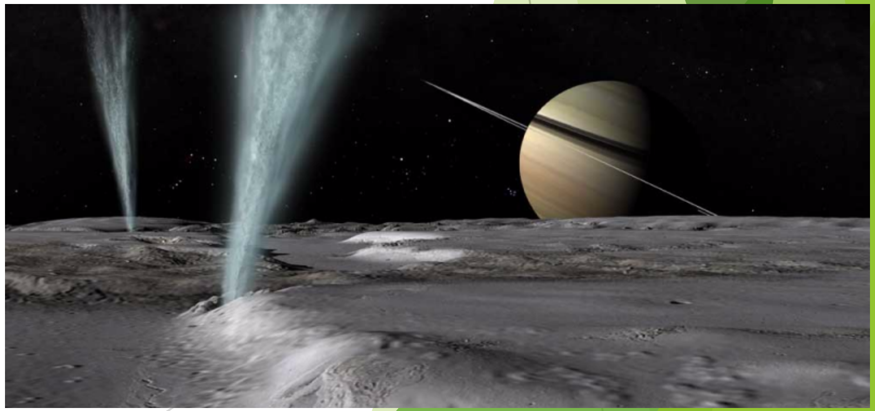

Stage 2: Furlagane Stage

The gas sublimates into solid vines and absorbs nutrients from the soil

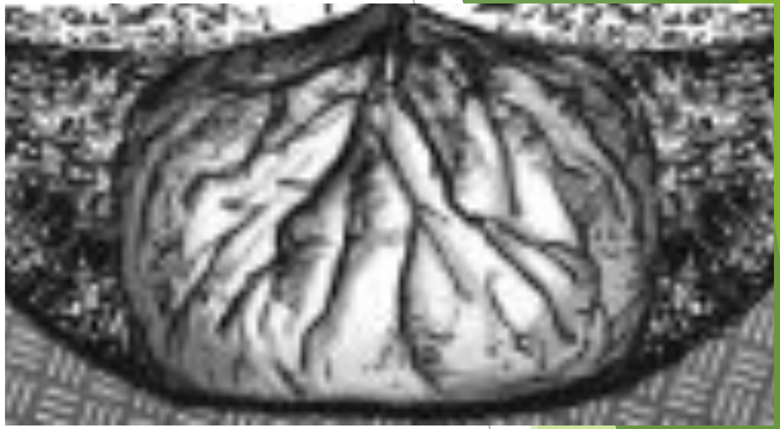

- Over time the **vapour will cool and sublimate, forming cellular tissue in a vine structure** around the walls of the burrow
- These **vines** then **absorb nutrients** from the surrounding soil
- These **nutrients are used in the development of more specialised cells which form the organs**
- Some of these **vines will later be used in the** development of their **skeletal system**

Stage 3: Levorg Stage

The vines use nutrients from the soil to form the first part of the brain

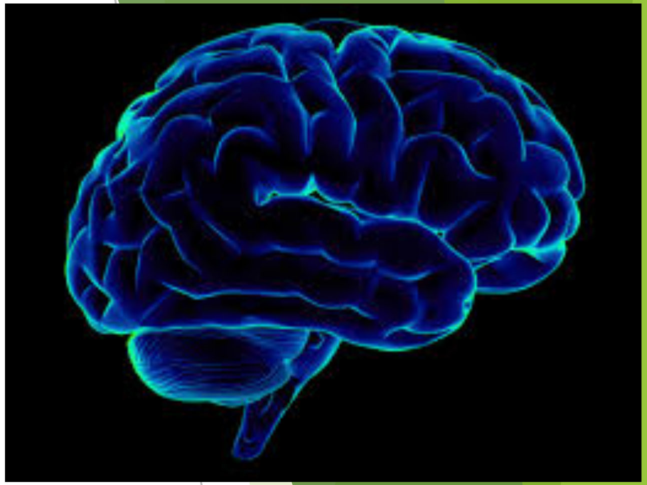

- The first organ to develop within the vines is the **Crixwin's primary neurological organ (ufrot)**
- This is **initially responsible for the formation of the other organs** (namely those used in the digestive and excretion systems), but is later also responsible for automatic processes such as the regulation of the Crixwin's epidermal respiratory system and motor control
- It is not until Stage 4 that what we might call "consciousness" develops - at this stage the ufrot acts in a similar way to the way Earth's microorganisms may behave - it serves only to direct the flow of vital nutrients and processes

Stage 4: Gresians Stages

The newly formed neurological organ (ufrot) continues directing nutrients from the soil into the construction of the skin and skeleton

- This is the **most pivotal** stage in a Crixwin's development as it sees them becoming sentient
- **Nutrients** from the soil are compounded by the vines to **form an epidermal tissue** which spreads across the surface of the kurin's walls and links all the vines into a comprehensive **"skin"**. The vines still remain in contact with the soil while the rest of the stage is completed
- **The ufrot then directs the flow of nutrients** to change the shape of the **vines** and compound the tissue into a more durable**, bone-like material called gritin.**

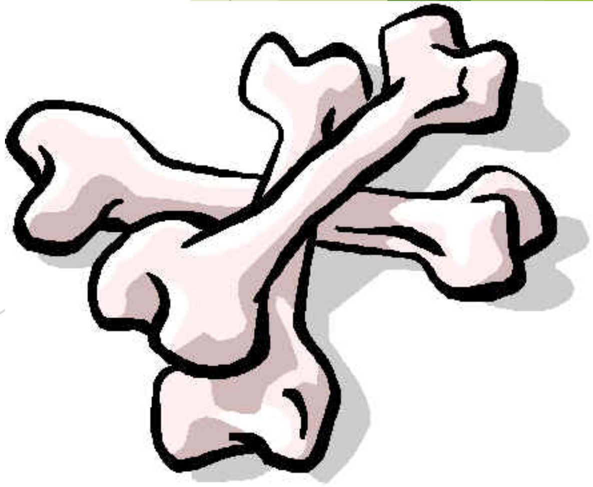

Stage 4 continued . . .

- As the gritin skeleton becomes integrated into the epidermis, the **ufrot** simultaneously **develops cortical areas responsible for proprioception**. From this initial proprioceptive ability, the rest of the Crixwin's consciousness and sensory systems will develop

- **Crixwin's are deaf but are able to sense vibrations to an incredibly sensitive degree** through the complex sensory network that runs through their epidermis - they can practically hear sounds that are loud enough and will normally feel the oncoming of any potential threats before we would even be able to hear it

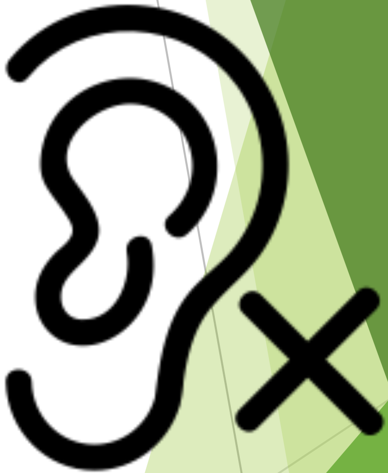

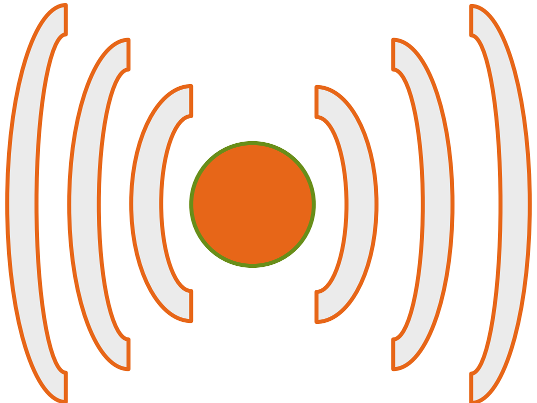

Stage 5: Anderlons Stage

The Crixwin's digestive and respiratory systems develop, and the vital Crixjuice is secreted into the Crixwin's interior cavity

- The **respiratory system of a Crixwin is found entirely within their epidermis** - so once the skeleton has completely formed this is the first organ to develop before the epidermis seals the gritin skeleton into position

- **The respiratory system is made up of small pockets scattered throughout the epidermis**. These pockets are called **irwigs** and will work to take in the atmosphere of Alun through the Crixwin's skin and **absorb any useful chemicals into the Crixjuice** which can then be used for important processes in the body

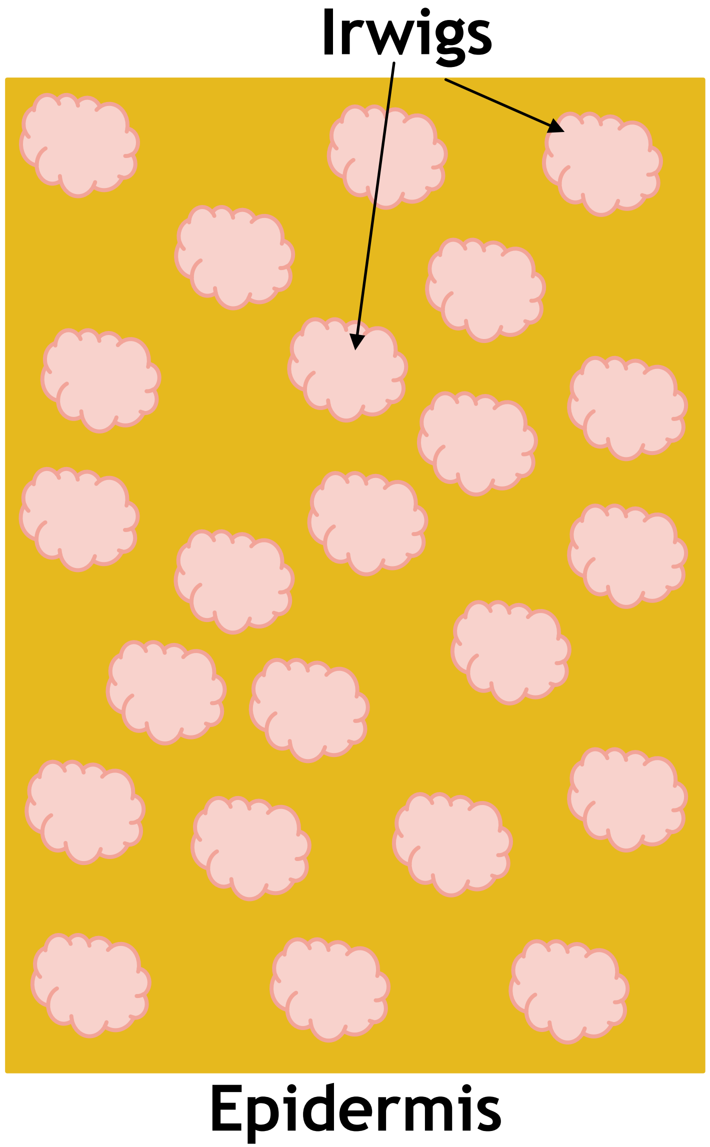

Stage 5 Continued . . .

- Once the **digestive system** (consisting of a **single sac with a selectively permeable membrane**) has formed and **successfully connected with the skeletal structure of the mouth** (which forms from a designated irwig being repurposed by the ufrot) the kurin serves its final purpose:

  - **The epidermis absorbs nutrients from the soil for the last time and produces the vitally important life-fluid known as Crixjuice**

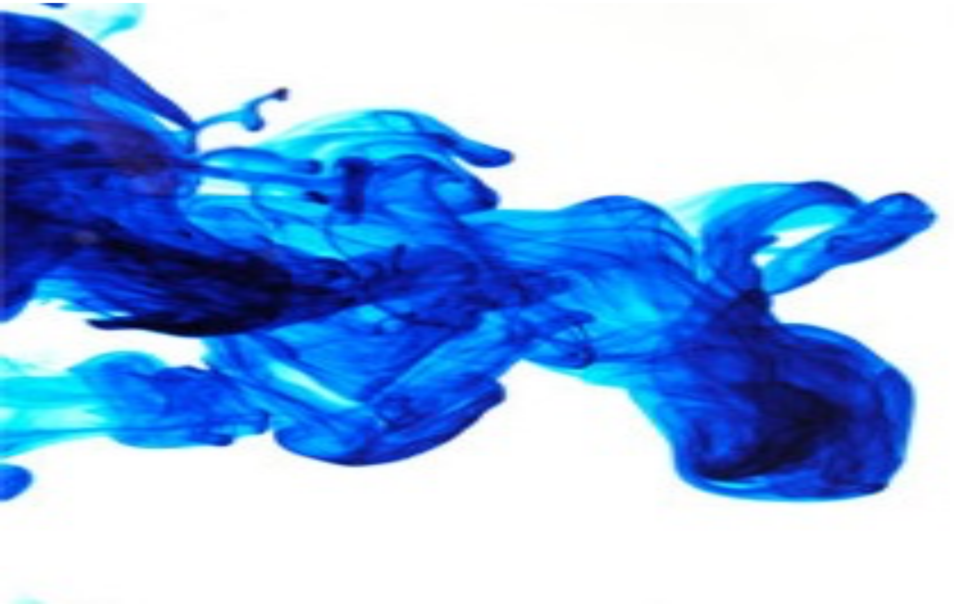

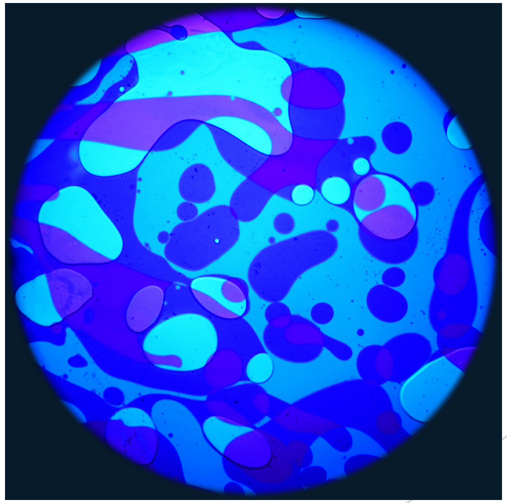

Stage 6: Impons Stage

The Crixwin emerges (albeit blind and without a mouth) from its kurin and its body undergoes the changes necessary for life on the surface

- With the body of the Crixwin being ready for survival on the surface, the **muscular and skeletal systems** make their **final major developments**
- A Crixwin's body is an **oblate spheroid shape** with a flattening ratio of around 0.35 on average, and moves **using four limbs with three digits each, with two retractable claws within each group of three**

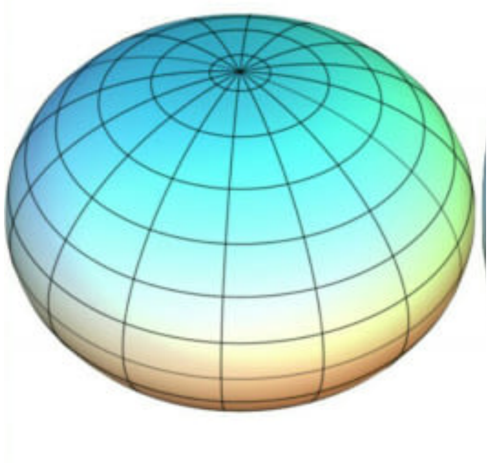

oblate spheroid

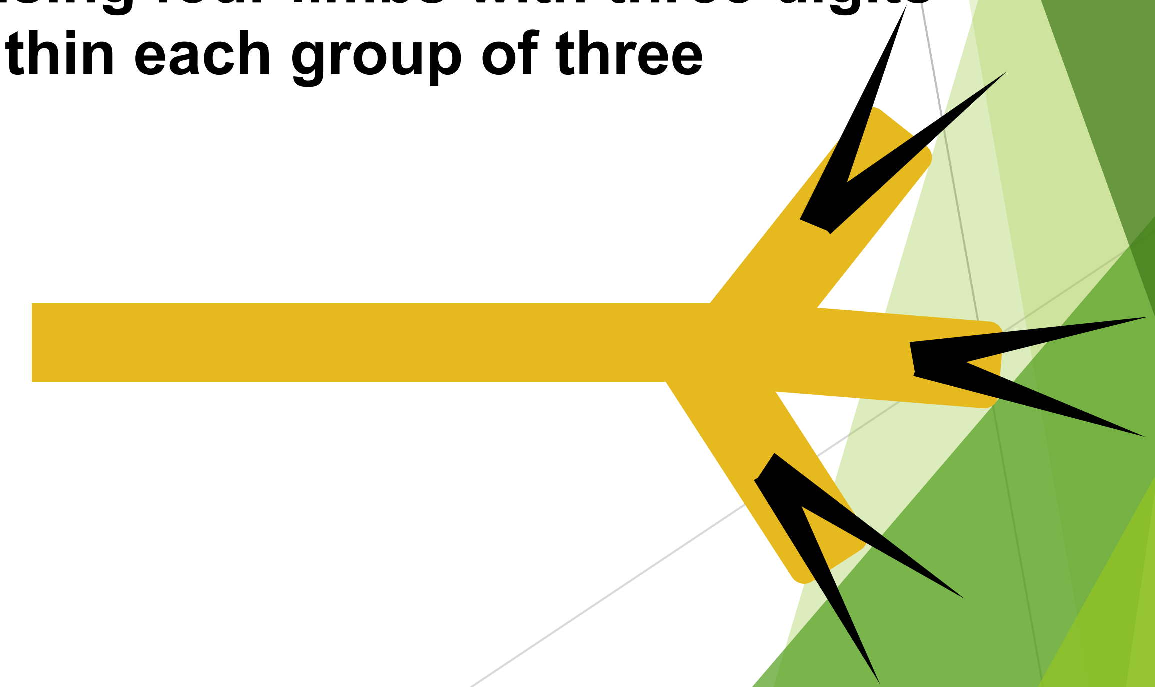

Stage 6 Continued . . .

- Each limb consists of two primary bones (and then more in each “hand”) which both utilise ball socket joints affording the Crixwin a great deal of **flexibility**

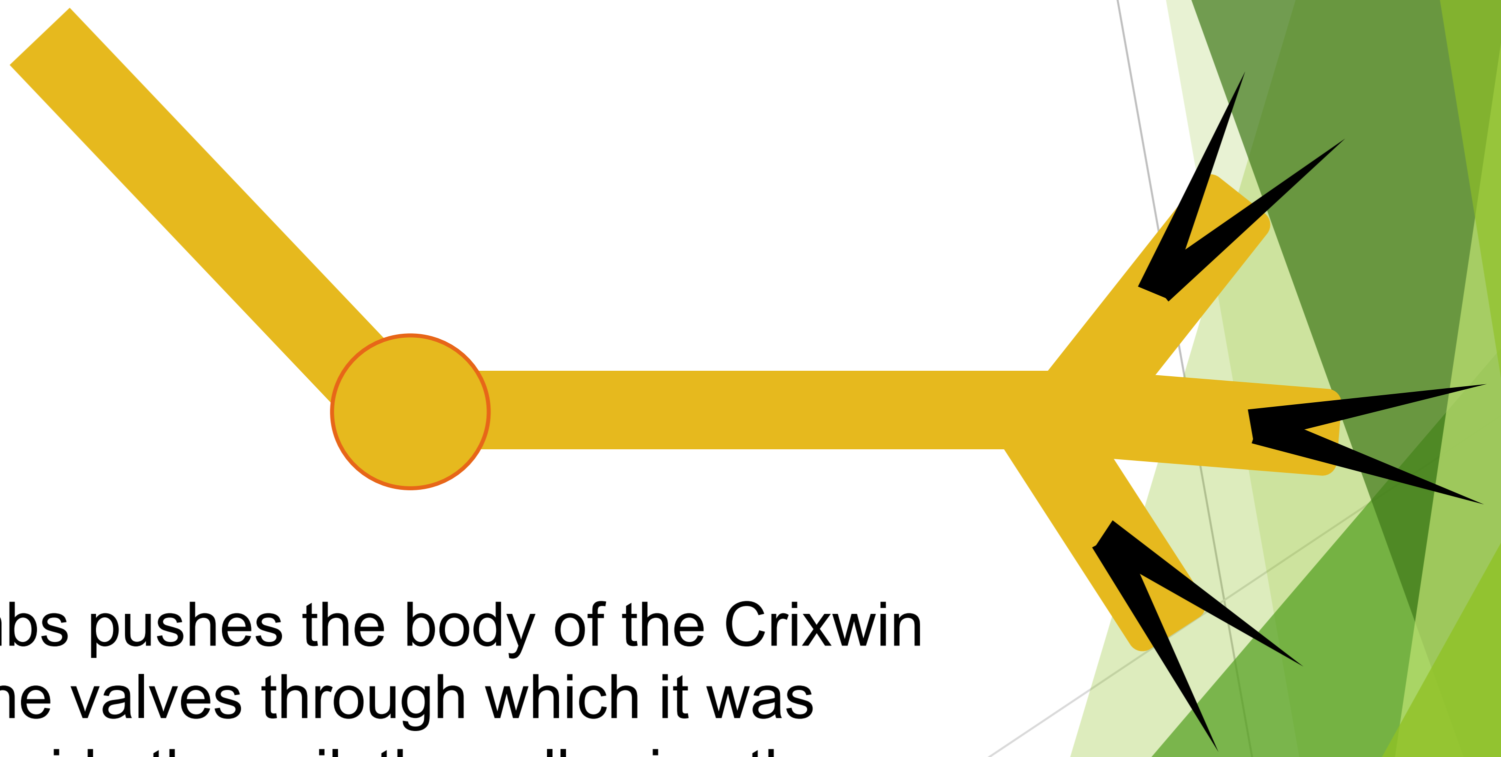

- The slow growth of these limbs pushes the body of the Crixwin out of the kurin, destroying the valves through which it was initially birthed and pushing aside the soil, thus allowing the complete development of the limbs

Stage 7: Borgavari Stage

The Crixwin's eyes and mouth develop, allowing it to see and eat, and marking the end of its biological development

- Once able to move around on the surface, the **Crixwin's ufrot directs the development of the mouth and eyes**

- The **mouth**, as previously mentioned, **develops from a pre-designated irwig**, growing teeth which are sharp enough to chew though the rough bark of the trees on the surface of Alun and get to the nourishing sap beneath

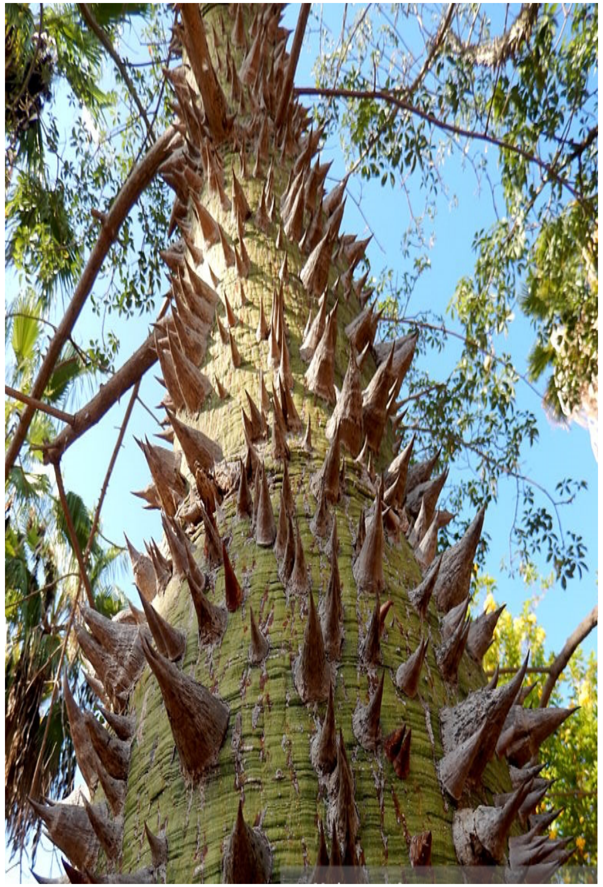

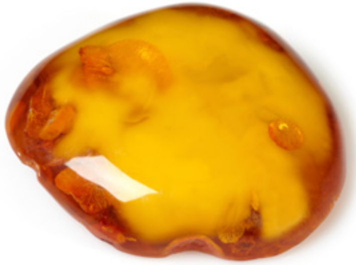

Stage 7 Continued . . .

- **The eyes then develop**, using two more **irwigs on the top and bottom of the Crixwin's body** - like the Earth chameleon, they are able to perceive depth without the need for two eyes looking at the same point (their eyes have a negative lens, positive cornea and monocular focusing abilities)

- The eyes are held on similar, albeit smaller, versions of the limbs which carry the Crixwins, meaning as well as being flexible in their motion they are also **able to see panoramically**

- **At this stage the Crixwin is fully biologically prepared for survival on the surface**

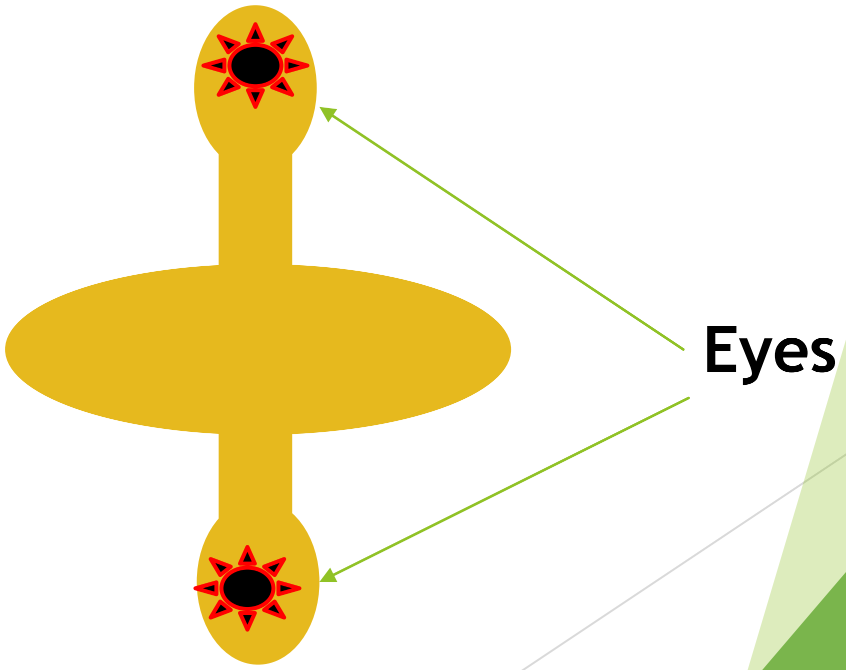

More about Crixwins

The Crixwin lives its life, learning more about its environment and building on the basic knowledge provided by the ufrot's subterranean development

- Due to the ufrot developing largely from the soil of the planet Alun, the Crixwin is already armed with some **biologically pre programmed knowledge of its environment**
  - The ufrot will already **have basic recognition of** some fundamental **elements which are toxic** to the Crixwin, and so if these elements are sensed by the sensory network of the Crixwin epidermis they will be avoided instinctively
  - However, in terms of which trees may yield the best sap, or which other creatures on Alun are suitable prey as examples, the Crixwin still has some development to go, in a similar way to how a human might come to learn the best way to perform a particular task

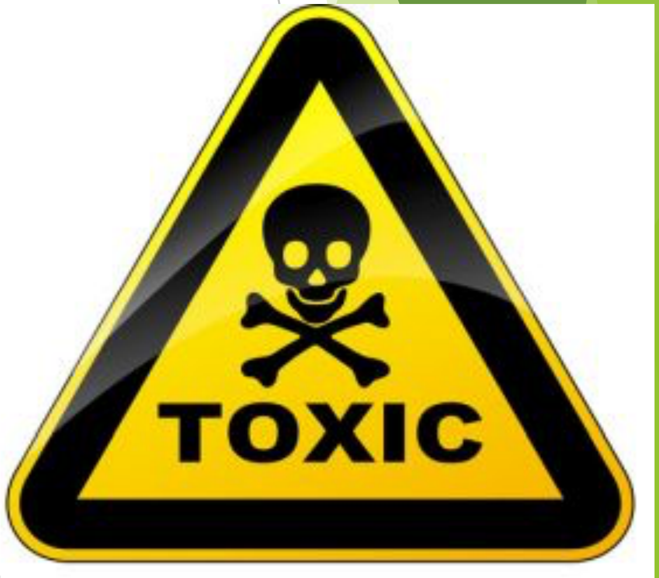

More about Crixwins

- **Crixwins do not need mates to reproduce.** The cycle of life starts again once the Crixjuice contained within the body has respired enough material and needs to excrete excess substances (which are the substances which make up the gas needed to create a new Crixwin)

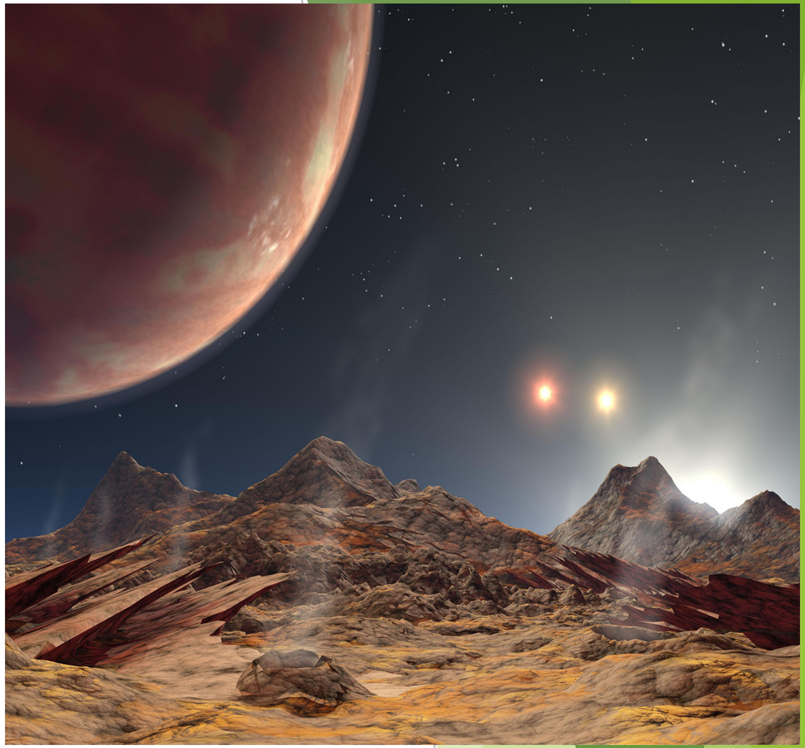

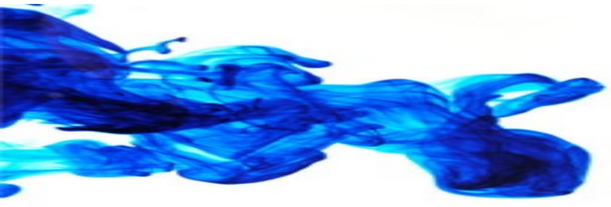

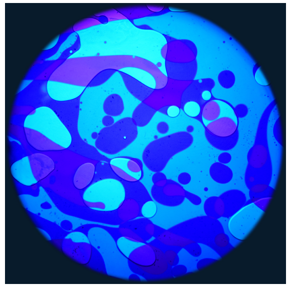

- Crixwins are **functionally immortal**, but there are plenty of other predators on the planet which keep Crixwin populations under control

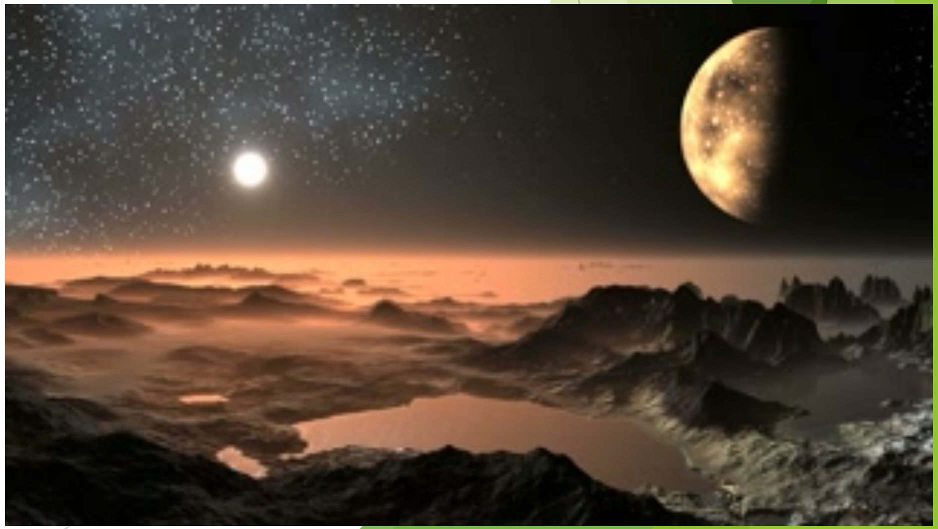

That's all you need to know about Crixwin's!

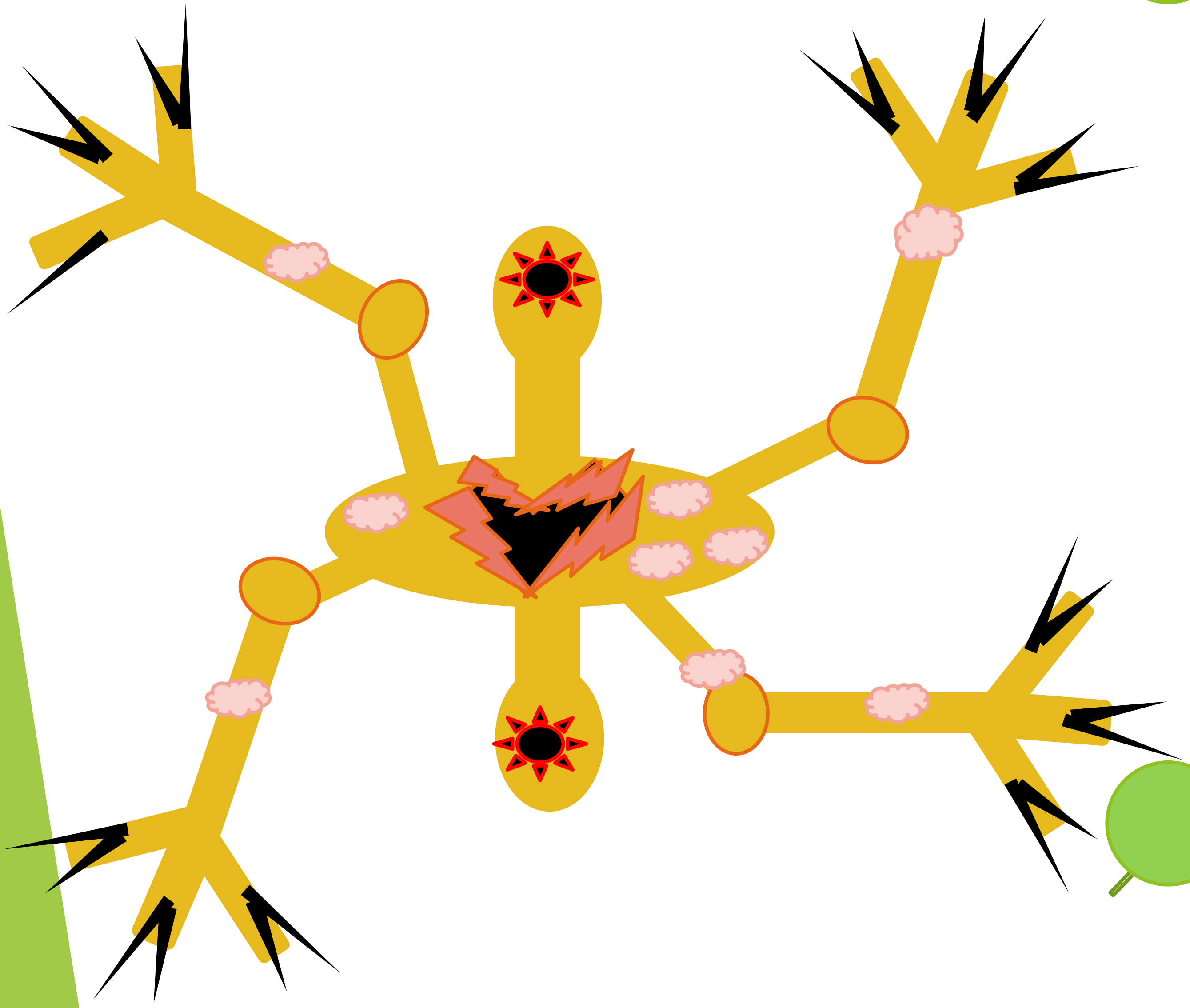

1: Negirans Stage
2: Furlagane Stage
3: Levorg Stage
4: Gresians Stage
5: Arderlons Stage
6: Impons Stage
7: Borgavari Stage
