# Supplementary material for: Immersive virtual environments and embodied agents for e-learning applications
Source: PeerJ Comput Sci. 2020 Nov 16;6:e315. doi: 10.7717/peerj-cs.315 (PMC7924662; doi:10.7717/peerj-cs.315)
Supplement: Supplemental Information 3 — The first section of the questionnaire contains the knowledge test, composed of 29 questions designed to test participants’ knowledge of the alien species. Three blocks of questions followed: learner satisfaction and engagement (7 items); learner motivation (3216items); and virtual presence (5 items). [file peerj-cs-06-315-s003.pdf]

Learning in VR

Survey Flow

Standard: Introduction (6 Questions)
Standard: Post-Learning Test (29 Questions)
Block: Personal satisfaction & engagement with the learning material (20 Questions)

Branch: New Branch
If
If Condition Non-Virtual Environment Is Not Selected

Standard: Virtual presence questions (6 Questions)
Standard: VR novelty questions (2 Questions)
Standard: Human Avatar Q's (1 Question)

EndSurvey:

Page Break

Start of Block: Introduction

Page Break

Q22 Participant Number

____

Q18 Condition

- ○ Non-Virtual Environment (1)
- ○ Virtual - No Tutor (2)
- ○ Virtual - Human Tutor (3)
- ○ Virtual - Block Tutor (4)

Q19 Age

____

Q20 Gender

- ○ Male (1)
- ○ Female (2)

Q55 Subject of Study

____

Q65 Do you have any learning disabilities which could impact your performance?

- ○ Yes (1)
- ○ No (2)
- ○ Prefer Not to Say (3)

Page Break

End of Block: Introduction

Start of Block: Post-Learning Test

Q67 Please complete the following test. Take your time and read the questions carefully. This test is negatively marked to discourage random guessing. Therefore, if you don't know the answer you can leave it blank. Once you have moved on from a question you won't be able to revisit the question. Good Luck!

Q54 **Arrange the following stages from 1 to 7** *(7 marks)*
(If you do not wish to answer this question leave the stages in the order presented, if you rearrange any of the stages the whole question will be marked)
_______ Impons stage (1)
_______ Gresians stage (2)
_______ Furlagane stage (3)
_______ Negirans stage (4)
_______ Arderlons stage (5)
_______ Borgavari stage (6)
_______ Levorg stage (7)

Q26 **What is the name of the planet that Crixwins inhabit?** *(1 mark)*

- ○ Arun (1)
- ○ Arilia (2)
- ○ Alun (3)
- ○ Kurin (4)

Q27 **After how many Earth months does a parent Crixwin secrete a gaseous substance into a cavern to begin the gestation period?** *(1 mark)*

- ○ 9 months (1)
- ○ 12 months (2)
- ○ 16 months (3)
- ○ 17 months (4)

Q28 **What is the name given to these caverns?** *(1 mark)*

- ○ Kurins (1)
- ○ Craters (2)
- ○ Ufrots (3)
- ○ Cavities (4)

Q29 **How do Crixwins find these caverns?** *(1 mark)*

____

____

____

____

____

Q30 **How are the caverns on the planet's surface created?** *(1 mark)*

○ Left behind when Crixwins emerge from the ground (1)

○ A type of flora (Gritin) form sacs of spores underground, once the spores are released, a crater is left behind. (2)

○ A type of flora (Lo'roon) form sacs of spores underground, once the spores are released, a crater is left behind. (3)

○ A type of flora (Gritin) cause vibrations which travel through the soil. (4)

Q31 **What forms once the gas secreted by the Crixwin cools and sublimates in the cavern?** *(1 mark)*

____

____

____

____

____

Q32 **What are the purposes of the vines which form when the gas cools?** *(2 marks)*

☐ They prevent the caverns from collapsing (1)

☐ They are used to absorb nutrients from the soil (2)

☐ They are what form the Crixwins organs (3)

☐ They are what form the Crixwins Skeleton (4)

Q33 **What is the name of the first organ that Crixwins develop?** *(1 mark)*

- Ufrot (1)
- Vines (2)
- Gritin (3)
- Epidermal tissue (Skin) (4)

Q34 **What is the initial function of the Crixwin's brain?** *(1 mark)*

- Regulation of the respiratory system (1)
- Motor Control (2)
- Consciousness (3)
- Directing the flow of vital nutrients and processes (4)

Q35 **In what developmental stage does a Crixwin develop consciousness?** *(1 mark)*

- Stage 7 (1)
- Stage 4 (2)
- Stage 5 (3)
- Stage 3 (4)

Q36 **Which is the most pivotal stage in a Crixwin's development?** *(1 mark)*

____

Q37 **How do Crixwin's detect sound?** *(1 mark)*

- ○ Sensory systems which are developed for hearing (1)
- ○ Proprioception (2)
- ○ They sense the vibrations (3)
- ○ They cannot detect sounds (4)

Q38 **What is used to form the skeleton AND what is the name of the skeleton?** *(2 marks)*

____

____

____

____

____

Q39 **What is secreted into the Crixwin's interior cavity?** *(1 mark)*

- ○ A gaseous substance (1)
- ○ Spores (2)
- ○ Irwigs (3)
- ○ Crixjuice (4)

Q40 **In what stage does the Crixwin's digestive and respiratory systems develop?** *(1 mark)*

____

Q41 **Where is the respiratory system located?** *(1 mark)*

____

Q42 **How does the Crixwin take in the atmosphere?** *(1 mark)*

- ◯ Through a selectively permeable membrane on the epidermis (1)
- ◯ Via large permeable sacs (irwigs) scattered throughout the epidermis (2)
- ◯ Via small pockets (irwigs) scattered throughout the epidermis (3)
- ◯ Via the Ufrot (4)

Q43 **What is the name given to the Crixwin equivalent of blood?** *(1 mark)*

____

Q45 **In what stage does the Crixwin's eyes and mouth develop?** *(1 mark)*

____

Q46 **How many digits does a Crixwin have?** *(1 mark)*

- ◯ 12 (1)
- ◯ 20 (2)
- ◯ 10 (3)
- ◯ 16 (4)

Q47 **From what do the mouth and eyes develop?** *(1 mark)*

- ◯ Irwigs (1)
- ◯ Vines (2)
- ◯ Epidermal tissue (3)
- ◯ Ufrot (4)

Q48 **At what stage does a Crixwin develop teeth?** *(1 mark)*

- ◯ Stage 8 (1)
- ◯ Stage 6 (2)
- ◯ Stage 7 (3)
- ◯ They do not have teeth (4)

Q49 **What do Crixwins eat?** *(1 mark)*

____

Q50 **What natural instincts do Crixwins possess?** *(1 mark)*

- ◯ Mating with other Crixwins (1)
- ◯ Which creatures are suitable prey (2)
- ◯ Avoidance of toxic material (3)
- ◯ Which trees yield the best sap (4)

Q51 **When do Crixwins reproduce?** *(1 mark)*

○ When they reach full maturity and mate with other Crixwins (1)

○ When a Crixwin is nearing the end of its life (2)

○ When Crixwins release spores from an irwig (3)

○ When the crixjuice contains excess nutrients (4)

Q52 **What is the lifespan of a Crixwin?** *(2 marks)*

____

Q53 **Arrange the following phrases into the correct order (From stage 1 to stage 8)** *(8 marks)*
(If you do not wish to answer this question leave the phrases in the order presented, if you rearrange any of the phrases the whole question will be marked)
______ The newly formed neurological organ (ufrot) continues directing nutrients from the soil into the construction of the skin and skeleton (1)
______ The Crixwin's digestive and respiratory systems develop, and the vital Crixjuice is secreted into the Crixwin's interior cavity (2)
______ The gas sublimates into solid vines and absorbs nutrients from the soil (3)
______ The Crixwin's eyes and mouth develop, allowing it to see and eat, and marking the end of its biological development (4)
______ Gas is secreted into a type of hole in the ground by a parent Crixwin (5)
______ The vines use nutrients from the soil to form the first part of the brain (6)
______ The Crixwin lives its life, learning more about its environment and building on the basic knowledge provided by the ufrot's subterranean development (7)
______ The Crixwin emerges (albeit blind and without a mouth) from its kurin and its body undergoes the changes necessary for life on the surface (8)

End of Block: Post-Learning Test

Start of Block: Personal satisfaction & engagement with the learning material

Q55 Please answer the following questionnaire about your personal satisfaction and engagement with the learning material, by rating how strongly you agree or disagree with the statements. Remember this questionnaire is subjective, there are no ‘right’ or ‘wrong’ answers.

Q2 **The learning experience captured my interest**

- ◯ Strongly disagree (1)
- ◯ Somewhat disagree (2)
- ◯ Neither agree nor disagree (3)
- ◯ Somewhat agree (4)
- ◯ Strongly agree (5)

Q3 **I found it easy to focus on the information provided**

- ◯ Strongly disagree (1)
- ◯ Somewhat disagree (2)
- ◯ Neither agree nor disagree (3)
- ◯ Somewhat agree (4)
- ◯ Strongly agree (5)

Q4 **The method of learning was appealing to me**

- Strongly disagree (1)
- Somewhat disagree (2)
- Neither agree nor disagree (3)
- Somewhat agree (4)
- Strongly agree (5)

Q5 **I was very absorbed in the learning experience**

- Strongly disagree (1)
- Somewhat disagree (2)
- Neither agree nor disagree (3)
- Somewhat agree (4)
- Strongly agree (5)

Q6 **I enjoyed the learning experience**

- Strongly disagree (1)
- Somewhat disagree (2)
- Neither agree nor disagree (3)
- Somewhat agree (4)
- Strongly agree (5)

Q7 **I put a lot of effort into learning the information**

- Strongly disagree (1)
- Somewhat disagree (2)
- Neither agree nor disagree (3)
- Somewhat agree (4)
- Strongly agree (5)

Q8 **It was important for me to learn effectively so that I could perform well on the test**

- Strongly disagree (1)
- Somewhat disagree (2)
- Neither agree nor disagree (3)
- Somewhat agree (4)
- Strongly agree (5)

Display This Question:

If Condition != Virtual - No Tutor

Q9 **The presence of the tutor increased my motivation to learn**

- Strongly disagree (1)
- Somewhat disagree (2)
- Neither agree nor disagree (3)
- Somewhat agree (4)
- Strongly agree (5)

Display This Question:

If Condition != Virtual - No Tutor

Q10 **The presence of the tutor was NOT distracting**

- Strongly disagree (1)
- Somewhat disagree (2)
- Neither agree nor disagree (3)
- Somewhat agree (4)
- Strongly agree (5)

Display This Question:

If Condition != Virtual - No Tutor

Q11 **I liked the tutor which was present in the learning experience**

- Strongly disagree (1)
- Somewhat disagree (2)
- Neither agree nor disagree (3)
- Somewhat agree (4)
- Strongly agree (5)

Page Break

Display This Question:

If Condition = Virtual - Human Tutor

Or Condition = Virtual - Block Tutor

Q60 I would prefer a tutor which was more realistic in terms of appearance

- Strongly agree (1)
- Agree (2)
- Somewhat agree (3)
- Neither agree nor disagree (4)
- Somewhat disagree (5)
- Disagree (6)
- Strongly disagree (7)

Display This Question:

If Condition = Virtual - Human Tutor

Or Condition = Virtual - Block Tutor

Q61 I would prefer a tutor which was less realistic in terms of appearance

- ☐ Strongly agree (1)
- ☐ Agree (2)
- ☐ Somewhat agree (3)
- ☐ Neither agree nor disagree (4)
- ☐ Somewhat disagree (5)
- ☐ Disagree (6)
- ☐ Strongly disagree (7)

Display This Question:

If Condition = Virtual - Human Tutor

Or Condition = Virtual - Block Tutor

Q62 I would prefer a tutor which was less realistic in terms of behaviour

- ☐ Strongly agree (1)
- ☐ Agree (2)
- ☐ Somewhat agree (3)
- ☐ Neither agree nor disagree (4)
- ☐ Somewhat disagree (5)
- ☐ Disagree (6)
- ☐ Strongly disagree (7)

Display This Question:

If Condition = Virtual - Human Tutor

Or Condition = Virtual - Block Tutor

Q63 I would prefer a tutor which was more realistic in terms of behaviour

- ☐ Strongly agree (1)
- ☐ Agree (2)
- ☐ Somewhat agree (3)
- ☐ Neither agree nor disagree (4)
- ☐ Somewhat disagree (5)
- ☐ Disagree (6)
- ☐ Strongly disagree (7)

Display This Question:

If Condition = Virtual - Human Tutor

Or Condition = Virtual - Block Tutor

Q68 I would prefer a tutor which was more realistic in terms of appearance

- ○ Strongly agree (1)
- ○ Agree (2)
- ○ Somewhat agree (3)
- ○ Neither agree nor disagree (4)
- ○ Somewhat disagree (5)
- ○ Disagree (6)
- ○ Strongly disagree (7)

Display This Question:

If Condition = Virtual - Human Tutor

Or Condition = Virtual - Block Tutor

Q69 I would prefer a tutor which was less realistic in terms of appearance

- ☐ Strongly agree (1)
- ☐ Agree (2)
- ☐ Somewhat agree (3)
- ☐ Neither agree nor disagree (4)
- ☐ Somewhat disagree (5)
- ☐ Disagree (6)
- ☐ Strongly disagree (7)

Display This Question:

If Condition = Virtual - Human Tutor

Or Condition = Virtual - Block Tutor

Q70 I would prefer a tutor which was less realistic in terms of behaviour

- ☐ Strongly agree (1)
- ☐ Agree (2)
- ☐ Somewhat agree (3)
- ☐ Neither agree nor disagree (4)
- ☐ Somewhat disagree (5)
- ☐ Disagree (6)
- ☐ Strongly disagree (7)

Display This Question:

If Condition = Virtual - Human Tutor

Or Condition = Virtual - Block Tutor

Q71 I would prefer a tutor which was more realistic in terms of behaviour

- ☐ Strongly agree (1)
- ☐ Agree (2)
- ☐ Somewhat agree (3)
- ☐ Neither agree nor disagree (4)
- ☐ Somewhat disagree (5)
- ☐ Disagree (6)
- ☐ Strongly disagree (7)

Display This Question:

If Condition = Virtual - No Tutor

Q64 I would prefer to have a tutor present in the learning environment

- ○ Strongly agree (1)
- ○ Agree (2)
- ○ Somewhat agree (3)
- ○ Neither agree nor disagree (4)
- ○ Somewhat disagree (5)
- ○ Disagree (6)
- ○ Strongly disagree (7)

End of Block: Personal satisfaction & engagement with the learning material

Start of Block: Virtual presence questions

Q25 Please answer the following questionnaire about how immersed you were in the environment and your feelings of virtual presence. Remember this questionnaire is subjective, there are no ‘right’ or ‘wrong’ answers.

Q12 **How engaged were all of your senses?**

- Not at All (1)
- Slightly (2)
- Moderately (3)
- Very (4)
- Completely (5)

Q13 **How aware were you of events occurring in the real world around you?**

- Not at All (1)
- Slightly (2)
- Moderately (3)
- Very (4)
- Completely (5)

Q14 **To what degree did your experiences in the virtual environment seem consistent with your real world experiences?**

- Not at All (1)
- Slightly (2)
- Moderately (3)
- Very (4)
- Completely (5)

Q15 **To what degree did you feel confused or disoriented at the end of the experimental session?**

- Not at All (1)
- Slightly (2)
- Moderately (3)
- Very (4)
- Completely (5)

Q16 **Were you involved in the experimental task to the extent that you lost track of time?**

- Not at All (1)
- Slightly (2)
- Moderately (3)
- Very (4)
- Completely (5)

End of Block: Virtual presence questions

Start of Block: VR novelty questions

Q56 Have you experienced Virtual Reality before taking part in this experiment?

○ Yes (1)

○ No (2)

Display This Question:

If Have you experienced Virtual Reality before taking part in this experiment? = Yes

Q57 Estimate how many times you have experienced VR (NOT including this tutorial)

○ 1-3 (1)

○ 3-5 (2)

○ 5-7 (3)

○ 10+ (4)

Page Break

End of Block: VR novelty questions

Start of Block: Human Avatar Q's

Display This Question:

If Condition = Virtual - Human Tutor

Q58 Did you notice anything odd about the human avatar? (if YES please specify)

- ○ Yes (1) ____
- ○ No (2)

End of Block: Human Avatar Q's
