# Supplementary material for: Immersive virtual environments and embodied agents for e-learning applications
Source: PeerJ Comput Sci. 2020 Nov 16;6:e315. doi: 10.7717/peerj-cs.315 (PMC7924662; doi:10.7717/peerj-cs.315)
Supplement: Supplemental Information 4 [file peerj-cs-06-315-s004.pdf]

Variable Information

Variable	Position	Label	Measurement Level	Role	Column Width
Participant_Number	1	<none>	Ordinal	Input	10
Condition	2	<none>	Nominal	Input	12
Gender	3	<none>	Nominal	Input	8
Age	4	<none>	Ordinal	Input	8
Subject	5	<none>	Nominal	Input	21
Learning_Disability	6	<none>	Nominal	Input	9
Test_Score	7	<none>	Scale	Input	11
Test_Percentage	8	<none>	Scale	Input	13
Satisfaction_engagement_Score	9	<none>	Scale	Input	8
Virtual_Presence_Score	10	<none>	Scale	Input	8
Tutor_Motivation_Score	11	<none>	Scale	Input	16
Q60	12	More realistic appearance	Nominal	Input	8
Q61	13	Less realistic apperance	Nominal	Input	8
Q62	14	Less realistic behaviour	Nominal	Input	8
Q63	15	More realistic behaviour	Nominal	Input	8
Q64	16	I would prefer a tutor	Nominal	Input	8
VR_Novelty	17	<none>	Nominal	Input	8
Q9	18	The presence of the tutor increased my motivation to learn	Scale	Input	13
New_Tutor_Motivation_Score	19	<none>	Scale	Input	15
Tutor_Likeability_Score	20	<none>	Scale	Input	8

Variable Information

Variable	Alignment	Print Format	Write Format
Participant_Number	Right	F8	F8
Condition	Right	F8	F8
Gender	Right	F8	F8
Age	Right	F8	F8
Subject	Right	F8	F8
Learning_Disability	Right	F8	F8
Test_Score	Right	F8	F8
Test_Percentage	Right	F8	F8
Satisfaction_engagement_Score	Right	F8	F8
Virtual_Presence_Score	Right	F8	F8
Tutor_Motivation_Score	Right	F8	F8
Q60	Right	F8	F8
Q61	Right	F8	F8
Q62	Right	F8	F8
Q63	Right	F8	F8
Q64	Right	F8	F8
VR_Novelty	Right	F8	F8
Q9	Right	F40	F40
New_Tutor_Motivation_Score	Right	F8	F8
Tutor_Likeability_Score	Right	F8	F8

Variables in the working file

Variable Values

Value		Label
Condition	1	Non-VR
	2	VR-Non
	3	VR-Human
	4	VR-Block
Gender	1	Male
	2	Female
Subject	1	Psychology
	2	EEE
	3	Maths
	4	Computer Science
	5	Mechanical Engineering
	6	Aeropsace Engineering
	7	TESOL
	8	Economics
	9	IME
	10	Chemistry
	11	Physics
	12	History
	13	Pharmacy
Learning_Disability	1	Yes
	2	No
	3	Prefer not to say
Q60	1	Strongly Disagree
	2	Disagree
	3	Somewhat Disagree
	4	Neitther agree nor disargree
	5	Somewhat Agree
	6	Agree
	7	Strongly Agree
Q61	1	Strongly Disagree
	2	Disagree
	3	Somewhat disagree
	4	Neither agree nor disagree
	5	Somewhat agree
	6	Agree
	7	Strongly Agree
Q62	1	Strongly Disagree
	2	Disagree

Variable Values

Value		Label
	3	Somewhat Disagree
	4	Neitther agree nor disargree
	5	Somewhat Agree
	6	Agree
	7	Strongly Agree
Q63	1	Strongly Disagree
	2	Disagree
	3	Somewhat Disagree
	4	Neitther agree nor disargree
	5	Somewhat Agree
	6	Agree
	7	Strongly Agree
Q64	1	Strongly Disagree
	2	Disagree
	3	Somewhat Disagree
	4	Neitther agree nor disargree
	5	Somewhat Agree
	6	Agree
	7	Strongly Agree
Q9	1	Strongly disagree
	2	Somewhat disagree
	3	Neither agree nor disagree
	4	Somewhat agree
	5	Strongly agree
